# A Theoretical Study of the Interaction of PARP-1 with Natural and Synthetic Inhibitors: Advances in the Therapy of Triple-Negative Breast Cancer

**DOI:** 10.3390/cimb46090558

**Published:** 2024-08-27

**Authors:** Albert Gabriel Turpo-Peqqueña, Emily Katherine Leiva-Flores, Sebastián Luna-Prado, Badhin Gómez

**Affiliations:** 1 Centro de Investigación en Ingeniería Molecular–CIIM, Universidad Católica de Santa María, Urb. San José s/n, Umacollo, Arequipa 04013, Peru; albert.turpo@ucsm.edu.pe (A.G.T.-P.); emily.bedregal@ucsm.edu.pe (E.K.L.-F.); 73615562@ucsm.edu.pe (S.L.-P.); 2 Facultad de Medicina Humana, Universidad Católica de Santa María, Urb. San José s/n, Umacollo, Arequipa 04013, Peru; 3 Facultad de Farmacia, Bioquímica y Biotecnología, Universidad Católica de Santa María, Urb. San José s/n, Umacollo, Arequipa 04013, Peru

**Keywords:** molecular mechanics, docking, molecular dynamics simulation, PARP-1

## Abstract

In the current study, we have investigated the secondary metabolites present in ethnomedical plants used for medicinal purposes—Astilbe chinensis (EK1), Scutellaria barbata D. Don (EK2), Uncaria rhynchophylla (EK3), Fallugia paradoxa (EK4), and Curcuma zedoaria (Christm.) Thread (EK5)—and we have compared them with five compounds of synthetic origin for the inhibition of PARP-1, which is linked to abnormal DNA replication, generating carcinogenic cells. We have studied these interactions through molecular dynamics simulations of each interacting system under physiological conditions (pH, temperature, and pressure) and determined that the compounds of natural origin have a capacity to inhibit PARP-1 (Poly(ADP-ribose) Polymerase 1) in all the cases inspected in this investigation. However, it is essential to mention that their interaction energy is relatively lower compared to that of compounds of synthetic origin. Given that binding energy is mandatory for the generation of a scale or classification of which is the best interacting agent, we can say that we assume that compounds of natural origin, having a complexation affinity with PARP-1, induce cell apoptosis, a potential route for the prevention of the proliferation of carcinogenic cells.

## 1. Introduction

In the current context, many endemic non-contagious diseases, such as cancer, have acquired an indisputable role as leading causes of death in humans [1]; we should also consider the progressive aging of the global population [2]. Therefore, identifying new therapeutic targets and designing non-invasive molecular mechanisms to inhibit and eliminate the growth of different types of cancer is an important objective [3]. Recently, various research groups have focused their attention on poly(ADP-ribose) polymerase 1 (PARP-1), which is a crucial element in DNA repair that offers promising possibilities for therapeutic intervention [4].

Among the various types of cancer, breast cancer (BC) is the leading cause of cancer-related death in women worldwide [5]. BC is the most common malignant tumor in women and represents 36% of oncological patients. The incidence of this malignant tumor is increasing in all the various regions of the world. Still, the highest incidence occurs in industrialized countries, which account for almost half of the cases worldwide [6]. This trend is mainly due to the so-called Western lifestyle, which is associated with poor diet, nicotinism, excessive stress, and little physical activity [7]. The definition of a new classification for breast cancer based on its gene expression pattern divided breast tumors into four “intrinsic subtypes”: the luminal subtype (Luminal A and Luminal B, characterized by the expression of the receptor gene estrogen), the HER2 subtype (characterized by the amplification of the HER2 gene), the basal subtype (a particular breast cancer that shows positivity for basal and myoepithelial markers, a lack of hormone receptors, and an amplification of the HER2 gene) and the “estrogen-like” subtype “Normal breast” (with a triple-negative phenotype but with cellular derivation typical of normal mammary epithelium). In particular, both basal and standard breast subtypes have already been recognized as being triple-negative (TNBC) [8].

TNBC breast cancer is a highly refractory and heterogeneous form of breast cancer [9]. TNBC breast cancer patients lack these selectable markers and have higher recurrence, resulting in higher mortality [3], while accounting for 24% of newly diagnosed breast neoplasms [10]. TNBC is characterized by a high risk of recurrence and metastasis and short progression-free survival and overall survival [11]. TNBC also tends to metastasize to different organs, including the lungs, liver, brain, bones, and others, leading to different treatment responses [12]. For this reason, it is essential to identify potential therapeutic agents aimed at the early clinical treatment of TNBC. Epidemiological investigations have found that about 10 to 40% of TNBC patients have mutations in breast cancer susceptibility gene 1 or 2 (BRCA 1, BRCA 2) [13], so this is considered an attractive topic for the scientific community in terms of the challenges that these breast cancer investigations entail [8], highlighting their therapeutic potential in specific clinical contexts.

PARPs are a family of DNA repair enzymes that transfer chains of ADP-ribose subunits to themselves or other proteins with negatively charged poly-ADP ribose (PAR) residues. Among the members of this family, PARP-1 is the primary cellular sensor of DNA damage [9], which it senses by adding PAR to the sites of single-stranded DNA damage (SSB), in addition to playing a fundamental role in the recognition and recruitment of DNA repair machinery [14]. Once DNA damage has occurred, PARP-1 rapidly binds to the damage site and recruits DNA repair (DDR) proteins through self-modified PAR chains, thus activating DNA repair mechanisms [15]. In addition to the SSB repair dominated by PARP, DNA double-strand fault (DSB) repair, dominated by homologous recombination repair (HRR), is crucial for genome stability [9]. HRR is a largely error-free pathway involving multiple steps; in this pathway, the dominant genes are BRCA 1 and BRCA 2 [16]. For cancers with BRCA 1 and BRCA 2 mutations, using PARP-1 inhibitors alone can promote a lethal accumulation of DNA faults, leading to the selective death of tumor cells [14]. In this case, PARP-1 inhibition significantly promotes DNA damage, a phenomenon known as “synthetic lethality” [17], due to defective DNA damage repair [18]. By definition, a synthetic lethal interaction between two genes occurs when perturbation of either gene alone is viable, but the perturbation of both genes simultaneously results in cell death [19].

PARP inhibitors are treatment options for a subset of TNBC with an HRR pathway [20]. For this reason, they are mainly used in breast tumors with defects in their BRCA 1 or BRCA 2 homologous recombination gene, in combination with agents that damage DNA, such as cis-platinum or topoisomerase-1 inhibitors [21]. Currently, in the pharmacopeia, Olaparib, Rucaparib, and Niraparib are the main drugs authorized by the US Food and Drug Administration (FDA). The mechanism of these inhibitors works through their binding to the catalytic domain of PARP-1, leading to reduced PARylation and, consequently, a defect in DNA repair [22]. However, some studies suggest that PARP-1 inhibitors also cause the formation of PARP1-DNA complexes with increased cytotoxicity and cell death [23]; on the other hand, some patients develop a resistance to PARP-1 inhibitors, which leads to poor treatment effectiveness [24]. Resistance to PARP-1 inhibitors is a problem that must be resolved to increase drug efficacy and patients’ chances of survival [25]. In the present study, we will address the interaction between PARP-1 and various naturally occurring and synthetic inhibitors to understand the factors governing their underlying molecular interactions. Exploring these interactions and their nature is necessary to develop more effective and specific therapeutic strategies. The diversity of these inhibitors, from compounds derived from natural sources to those designed synthetically, will give us a greater understanding of the processes involved [26].

## 2. Computational Details

The structure of the PARP-1 protein used in the present investigation was taken from the Protein Data Bank (PDB) server [27] and has the identification code ID: 4DQY. The file downloaded from the server contained additional molecules that facilitated its crystallization, so a clean structure was obtained. The resulting structure had missing residues. The Alphafold server [28] was used to obtain a complete structure. Because Alphafold only predicts protein structures without considering metal ions, the zinc fingers’ zinc atoms were manually placed in two structures. The secondary metabolites to interact with the protein were selected through a bibliographic search in databases such as Web of Science, Scopus, PubMed, and the National Center for Biotechnology Information (NCBI) using key terms and their combinations, such as “Natural products” (Natural products), “Synthetic products”, “Breast cancer”, “Inhibitor”, “PARP-1”, “MCF-7”, “MDA-MB-231”, and “BT-474”. The results obtained were grouped into two categories: those of natural origin and synthetic ones. The naturally occurring secondary metabolites selected were (3S,3aS,5S,6R,8aS)-5-isopropyl-3-methyl-8-methyleneoctahydro-6H-3a,6- epoxyazulen-6-ol or Curcumol (EK5) [29], methyl (E)-3-methoxy-2-((2S,3R,12bR)-3-vinyl-1,2,3,4,6,7,12,12b-octahydroindolo[2,3-a]quinolizin-2-yl)acrylate or Dehydrocurvularin (EK4) [30], (S,E)-11,13-dihydroxy-4-methyl-4,5,6,7-tetrahydro-2H-benzo[d][1]oxacyclododecine-2,10(1H)-dione or Hirsutein (EK3) [31], (1R,2S, 3R,4R,4aS,8aR)-3-hydroxy-3,4,8,8a-tetramethyl-4-((E)-2-(5-oxo-2,5-dihydrofuran-3- yl)vinyl)-1,2,3,4,4a,5,6,8a- octahydronaphthalene-1,2-diyl dinicotinate or Escutebarbatin A (EK2) [32], and 3B,6B-dihydroxides-12-en-27-oic acid (EK1) [33]. Additionally, the selected metabolites of synthetic origin were 1-(5-(3-(Benzofuran-2-yl)-1-phenyl-1H-pyrazol-4-yl)-4,5-dihydro-3(1H-pyrrol-2-yl)pyrazol-1-yl)ethanone (DK1) [34], 2-(3,4-Dimethoxybenzyl)-5-(3-(2-fluoro-3-methylpyridin-4-yl)phenyl)-1,3,4oxadiazol (DK5) [35], $3^{\prime }$-(5-(4-Methoxybencil)-1,3,4-oxadiazol-2-yl)-[1,$1^{\prime }$-biphenyl]-3carbaldehyde (DK2) [35], 2-[2-(4-hydroxy-phenyl)-vinyl]-3H-quinazolin-4-one (DK3) [9], and 6-(3,5-Dimethyl-1H-pyrazol-1-yl) sulfonyl)-1,4-dihydroquinoxaline-2,3dione (DK4) [36]. Eleven bioactive secondary metabolites were considered that will act as ligands in the protein complex, of which only [34,35,36,37] had to be constructed using the ChemDraw program [38], while the rest [29,30,31,32] were obtained from the PubChem server [39]. For all secondary metabolites, a force field was constructed according to their OPLS-AA functional [40,41] using the LigParGen server [42]. Likewise, in all the secondary metabolites, their charges were recalculated using the Hirshfeld approximation [43] and using the Gaussian 16 program [44] at the quantum level with the CAM-B3LYP functional [45] and the basis function TZVP [46,47].

The structure of PARP-1 was solvated (TIP3P) [48] and neutralized under physiological salt ion conditions. To bring it to equilibrium, we used canonical assembly (NVT) to introduce a thermostat at a physiological temperature of 309.65 K for a period of 10 ns. We introduced pressure using the Isobaric Isothermal assembly, and the trajectory used was 500 ns, all using the GROMACS computer package [49,50] and using OPLS-AA force field. Since this computational package uses stochastic approximations, we used the resulting structure as input for the Autodock Vina [51,52] software (https://vina.scripps.edu/, accessed on 2 June 2024). To perform molecular docking, we put the protein in a box with 150 Å dimensions per side and centered it at the midpoint of the box. The box has an energy range of three, an exhaustiveness of eight, and a total number of modes of 10 per repetition; we performed 2000 repetitions for each ligand in blind conditions, using the energy criterion to select the best-interacting structure.

We proceeded to subject all the interacting complexes to a molecular dynamics simulation using the GROMACS computational package; in the same way as with the structure of PARP-1, the complexes were solvated with TIP3P-type water molecules and neutralized to physiological saline ion conditions. Their protein–ligand complexes were placed in the center of a box with 1 nm between the system’s surface and the box’s edge. An earlier step in the molecular dynamics simulation was to minimize the forces involved in each system. The temperature was introduced using the V-rescale thermostat [53] and considering the physiological temperature of 309.65 K; in this step, a molecular simulation was carried out for 10 ns. Pressure was introduced using the Parrinello-Rahman barostat [54,55,56] and with a trajectory of 100 ns.

The resulting trajectories were analyzed to demonstrate that they were in their equilibrium region; for this, we obtained the root mean square deviation (RMSD), root mean square fluctuation (RMSF), and radii of gyration (RG). The visualization was carried out using UCSF Chimera software (https://www.cgl.ucsf.edu/chimera/, accessed on 2 June 2024) [57], where the data on the connection of the protein–ligand biomolecular assemblies could be seen. The graphs were plotted using GNUPlot software (http://www.gnuplot.info/, accessed on 2 June 2024) [58], and the LigPlot server [59] was also used, which provided us with 2D representations of the electrostatic and hydrophobic interactions between the protein and the ligands. On the other hand, electrostatic surfaces were generated by the Adaptive Poisson–Boltzmann Solver (APBS) approach [60,61], using its online server to better understand the nature of the interactions.

Finally, we calculated the interaction free energies using the molecular mechanics Poisson–Boltzmann surface area approximation (MM/PBSA) [62,63] to obtain inhibition energies.

## 3. Results and Discussion

**Figure 1:**
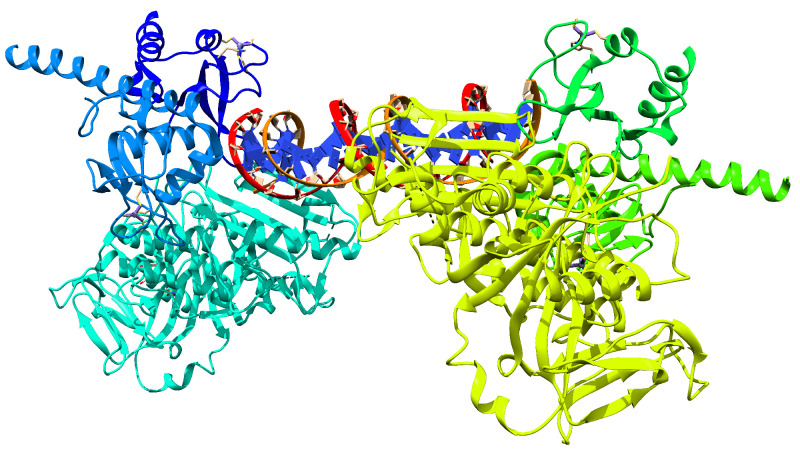
Structure of the PARP-1 protein obtained from the PDB server, ID: 4DQY.

The structure of PARP-1 that was found in the PDB database was 4DQY, which had six chains and a DNA segment, as well as two zinc atoms in each trimer, as we can see in Figure 1. Figure 1 additionally contained ethylene glycol structures that were used for crystallization.

**Figure 2:**
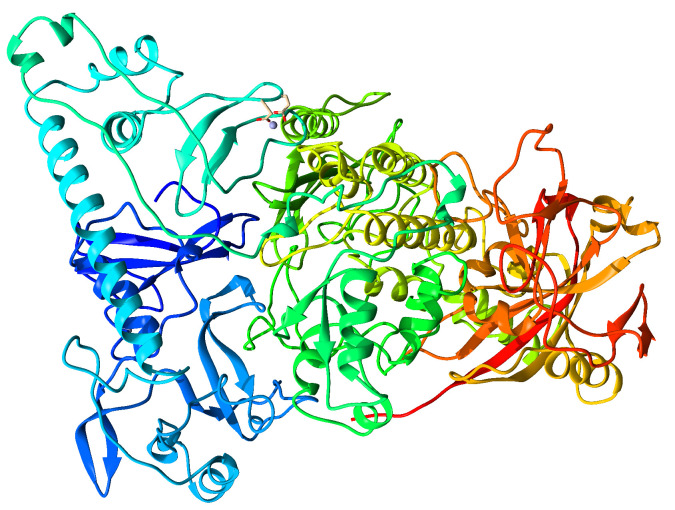
Initial secondary structure of PARP-1 (ID: 4DQY), without additional structural agents used for crystallization.

The DNA chain segment and the ethylene glycol molecules were removed using UCSF-Chimera software; the chosen trimer had missing residues on each of its chains, which had to be completed using the Alphafold online server. The clean and complete structure is presented in Figure 2, which contains two zinc atoms, generally known as zinc fingers, since these give the metalloprotein its functionality.

**Figure 3:**
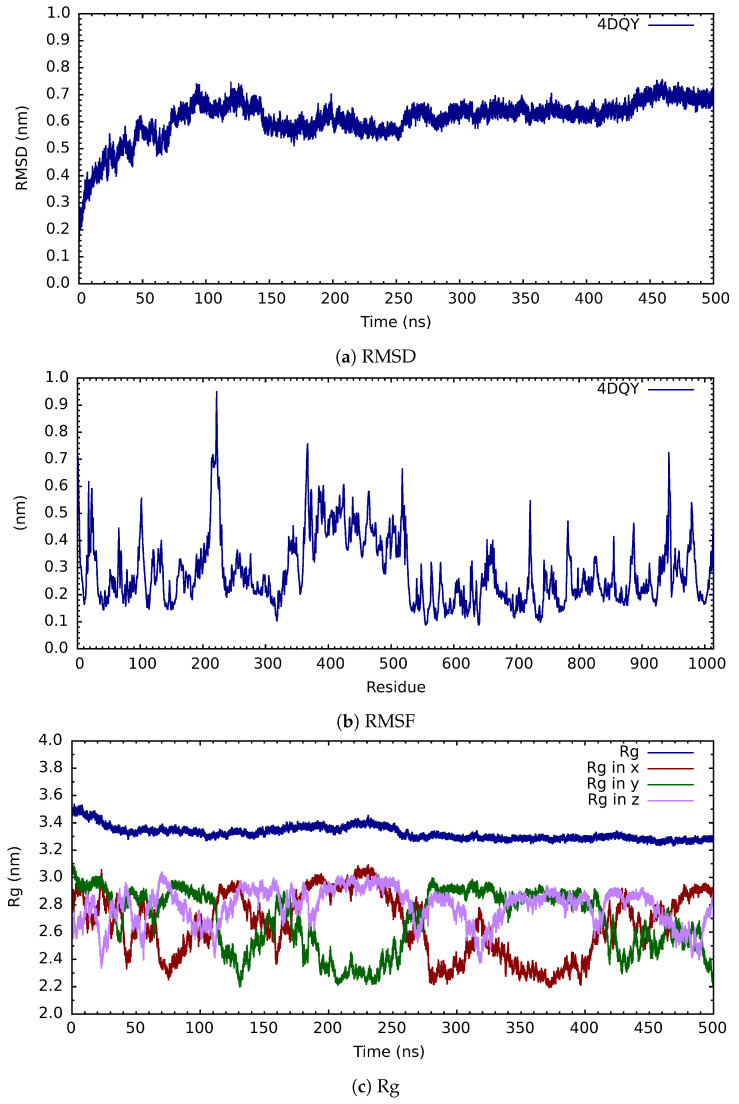
Analysis of structural parameters RMSD, RMSF, and Rg of PARP-1 after 500 ns of molecular dynamics simulation.

The clean structure of PARP-1 (ID:4DQY), which contained a total of 1014 residues and 2 zinc atoms, was placed in a cubic box with dimensions of 13.4414 nm per side; in this space, its solvation was carried out. Through the use of the Gromacs computational package, and making use of the TIP3P water model, neutralization was likewise carried out with a total of twenty-six chlorine atoms, which indicates that the total charge of the system was twenty-six positive (+26) due to its physiological pH; therefore, only sodium and chlorine salt ions were added, with the total amount added being four hundred and fifty-four (454), which corresponds to a concentration of 0.15 M. This resulted in a total of seventy-four thousand four hundred and eighty-two water molecules (74,482) in the box. With this system, the forces were minimized using the steep algorithm and an energy tolerance level of 1.0 kJ/mol. After this step, the temperature was introduced for 10 ns; these final files allowed us to start the molecular simulation dynamics for a period of 500 ns, during which the pressure was introduced as the last variable. A stability analysis of the cone structure was carried out on the final files of the molecular dynamics simulation in terms of RMSD, RMSF, and the radius of gyration; these are presented in Figure 3.

When we analyze the root mean square deviation of distances (see Figure 3a), there is an empirical rule that when the structure moves within a delta of 0.2 nm, we are in the equilibrium region, so we observe that from 250 ns onwards, our PARP system-1 (4DQY) is in equilibrium. From the graph of the root mean square deviation of the fluctuation of the residuals (see Figure 3b) we can say that, during the trajectory, the regions that have presented the most significant movement or fluctuation are between the residuals 200 and 240. The area between residues 360 and 530 also presents an appreciable fluctuation, as does that between residues 920 and 990. Finally, when we see the total radius of gyration (see Figure 3c), we can observe that the system has entered a compaction process from the beginning; only in the region of 100 ns to 250 ns does it move into an expansion process and then continue its compaction process. When we analyze the components of the radius of gyration, on the x-axis, the system contributes more to the expansion process in the region above. In contrast, the y-axis contributes the most to compaction.

**Figure 4:**
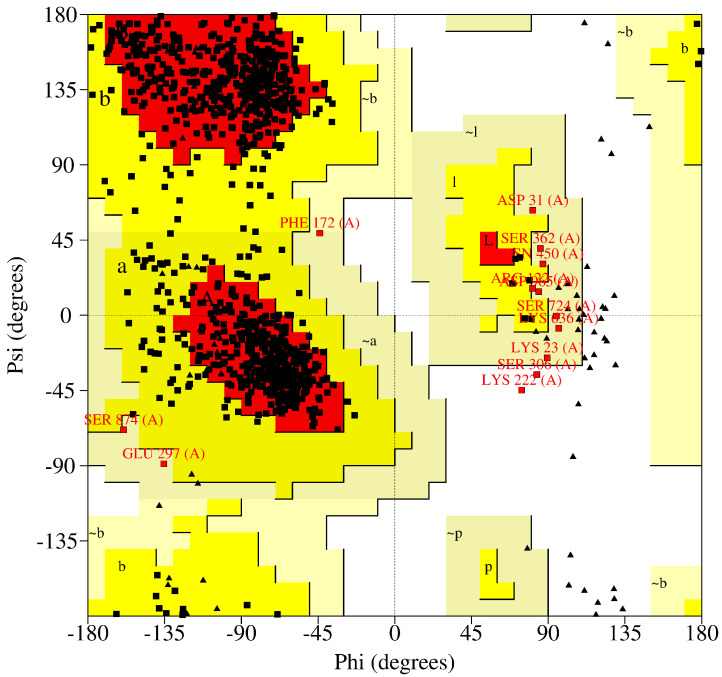
Ramachandran diagram analysis of PAR-$ after the 500 ns simulation.

Likewise, we carried out an analysis of the Ramachandran diagram (see Figure 4) to be able to use empirical rules to know whether our structure occurs naturally. From the results, we observe that we have a total of seven hundred forty-eight (748) residues that are found in favored regions (83.4%), while in the allowed areas we found a total of thirty-six (136) residues (15.2%) and a total of eleven (11) residues were found in the generously allowed regions (1.2%); this makes a total of eight hundred ninety-seven (897) which is 99.8% of the residues, with only two residues (2), which represent 0.2%, found in disadvantaged regions. This entire analysis was carried out excluding glycines, several prolines (4), several preprolines (44), and several glycines (71).

**Figure 5:**
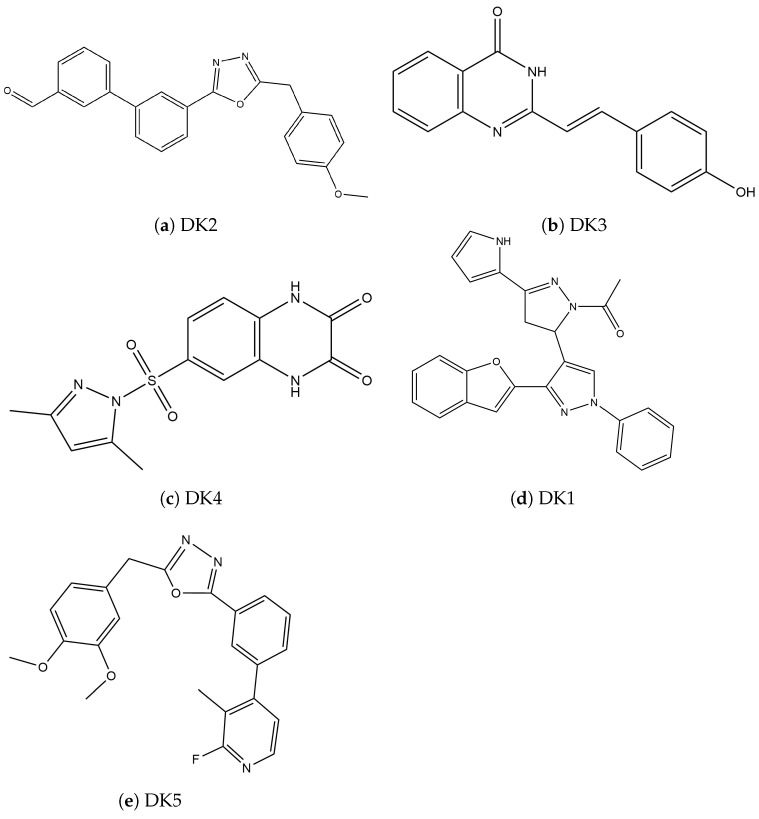
Two-dimensional structure of inhibitors of synthetic origin.

Next, we searched for inhibitory compounds of synthetic origin that may have a study-stage pharmacological license; we found five (05 compounds), which are presented in Figure 5.

**Figure 6:**
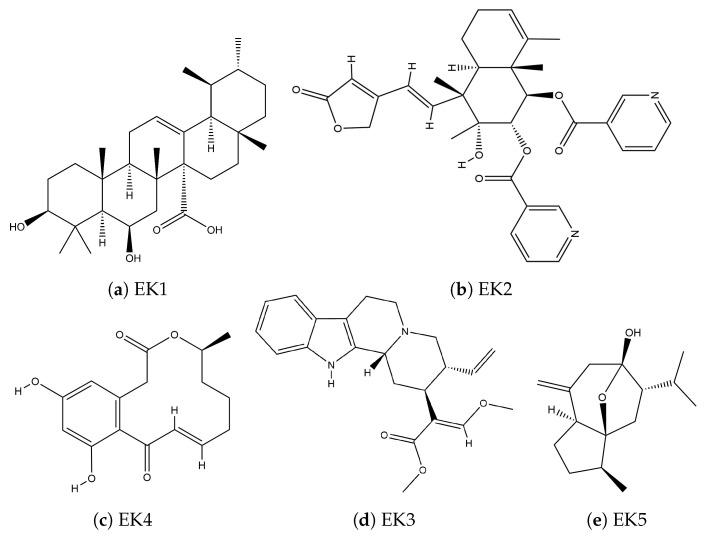
Two-dimensional structure of inhibitors of natural origin.

The structures of the compounds of natural origin found in the databases that are potential PARP-1 inhibitors are presented in Figure 6 in a two-dimensional (2D) representation; unlike those of natural origin, synthetics maintain a functional pattern, while those of natural origin present completely different functional forms.

By using the Autodock Vina computational package, the coupling of all compounds of natural and synthetic origin with the stabilized structure of PARP-1 was carried out; for each, a total of two thousand (2000) events were carried out due to the stochastic nature of the coupling approximation. The one with the lowest energy was chosen for each set of two thousand (2000) systems, as seen in Table 1. The values that AutoDock Vina gives us for these interaction energies are referential, because the force field only considers geometric parameters derived from Van der Waals volumes.

Analyzing the results of the binding energies, we can say that inhibitors of synthetic origin have better binding energy. Within the group of synthetic compounds, DK5 has the best interaction energy of −10.260 kcal/mol. In comparison, the best compound of natural origin is EK1, which has an interaction energy of −9.997 kcal/mol. For practical purposes, the difference in binding energy between the best synthetic compound and the best natural compound is only 0.263 kcal/mol; this value is within the margin of experimental error, so this could indicate that both have practically the same affinity. It should be noted that since this is a reference energy in the gas phase, it only provides us with the potential structures that could present the best interaction using the volume approximations of the Autodock Vina program. To identify the binding energy adequately, we proceeded to simulate the molecular dynamics of the system under physiological conditions for a period of 100 ns.

After performing the molecular dynamics simulation, we analyzed the interaction energy of each system’s last 10 ns. These results are presented in Table 2, so we can see that the interaction energy of the synthetic systems is in the following order: DK1 > DK2 > DK4 > DK5 > DK3, while the order of the values of the interaction energy of the compounds of natural origin are EK2 > EK3 > EK1 > EK5 > EK4. We can observe that there is a difference of approximately 11.626 kcal/mol between the natural systems and the synthetic ones.

**Figure 7:**
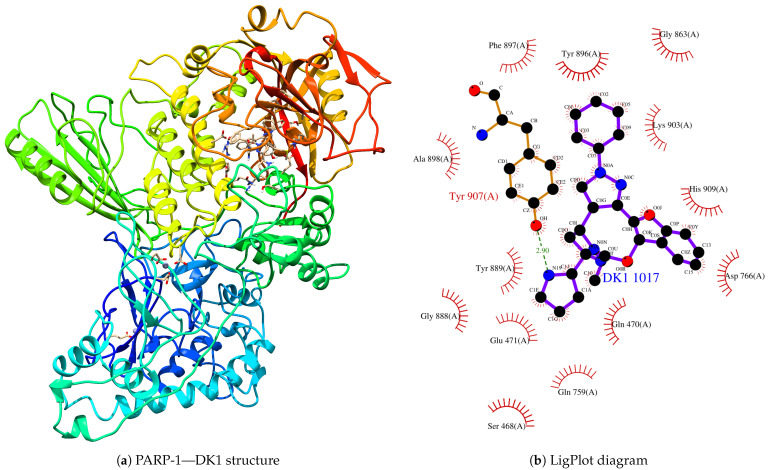
(**a**) Final structure of the PARP-1—DK1 coupled system after conducting a molecular dynamics simulation. (**b**) LigPlot diagram of the interaction.

From the final structures of the interacting systems, we analyzed the interaction zones of the synthetic and natural origin compounds, using the LigPlot server to identify the amino acid residues linked to the interaction processes. Thus, the synthetic compound that interacts best with PARP-1 is DK1, as shown in Figure 7.

From the PARP-1—DK1 structure, it can be observed that the zinc fingers have been maintained in this structure. Still, the DK1 compound is found in the cavity generated by the tertiary structure of the protein, thus presenting us with a structural satisfactory quaternary. When analyzing the interactions using the LigPlot server, we can see that Tyr-907, through the hydroxyl group, is the region closest to the compound, with a distance of approximately 2.93 A. Likewise, the following amino acids surround the compound: Ser-468, Gln-470, Glu-471, Gln-759, Asp-766, Gly-863, Gly-888, Tyr-889, Tyr-896, Phe-897, Ala-898, Lys-903, Tyr-907, and His-909.

**Figure 8:**
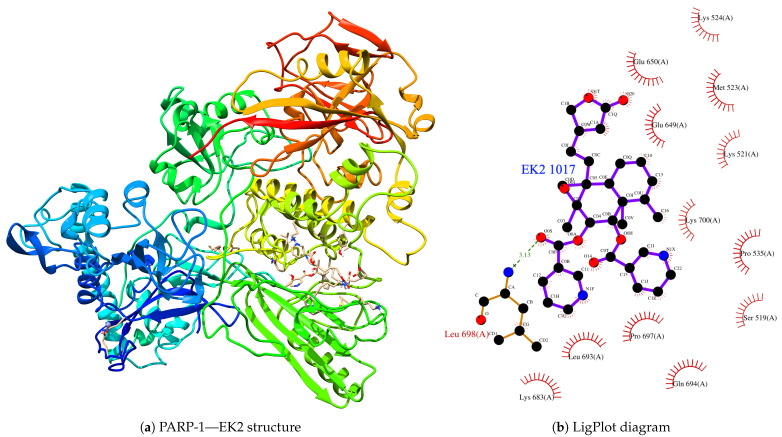
(**a**) Final structure of the PARP-1—EK2 coupled system after conducting a molecular dynamics simulation. (**b**) LigPlot diagram of the interaction.

In terms of the inhibitors of natural origin, their complex system is presented in Figure 8. When we performed its analysis using LigPlot to determine the closest residues in the interaction process, we found that Leu-698 is the best approach that presents a complex. Still, we found that a total of thirteen (13) amino acid residues interact with the PARP-1 protein; these are Ser-519, Lys-521, Met-523, Lys-524, Pro-535, Glu- 649, Glu-650, Lys-683, Leu-693, Gln-694, Pro-697, Leu-698, and Lys-700. From the classification of the residues, we can demonstrate that the interaction cavity is fundamentally polar.

**Figure 9:**
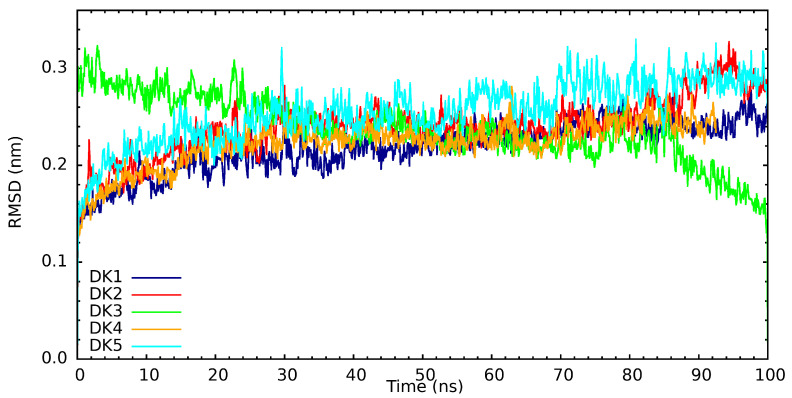
RMSD for the tnteraction of PARP-1 with synthetic origin compounds.

When performing an analysis of the root mean square deviation of the distances (RMSD) of the interaction between the protein and the synthetic compounds of interest (see Figure 9), it is essential to remember that, empirically, they are considered to be in the equilibrium region when they are within a delta of 0.2 nm. We can observe that DK1 reaches the equilibrium region after 30 ns of simulation and remains there during the rest of the simulation trajectory. Similarly, we can observe that DK2, DK4, and DK5 processes’ arrival at the equilibrium region is very similar; only in the case of the interaction with DK3 can we observe that its RMSD continues to decrease gradually, and this when observing the three-dimensional image. Here, we observe a gradual distancing of the compound as it is corroborated, with an absolute decrease in the interaction’s energy.

**Figure 10:**
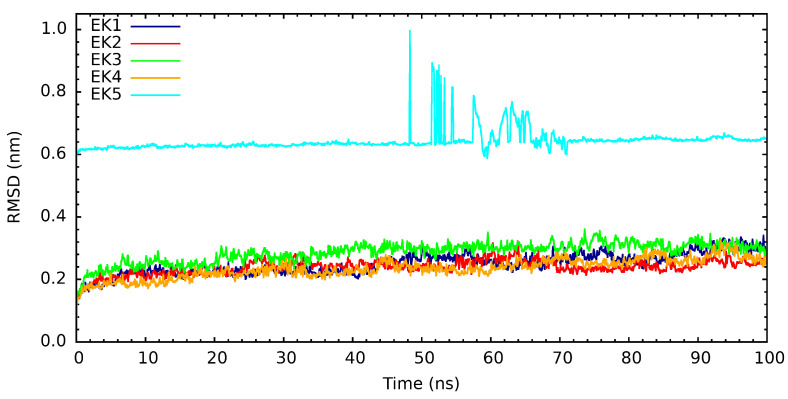
RMSD for the interaction of PARP-1 with natural origin compounds.

In the case of the root mean square deviations (RMSDs) of the interaction between PARP1 and compounds of natural origin, these graphs can be observed in Figure 10. These graphs show that EK5 presents strange behavior between 46 ns and 72 ns. When observing the structures in that simulation period, we realize that the compound moves away from the PARP-1 protein, and, after 72 ns, it approaches it again. The trajectory region of the MMPBSA analysis only considers the last 10 ns, so this behavior should not affect the interaction energy calculation. In terms of the other cases, we can observe that after the initial 20 ns, these systems enter an equilibrium region in all cases, and we highlight EK2 because it presents a lower fluctuation scale.

**Figure 11:**
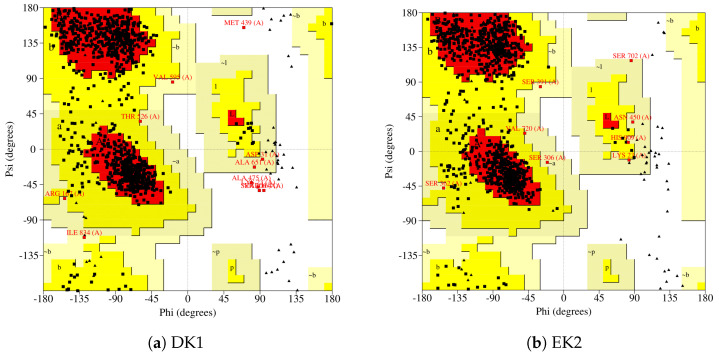
Ramachandran plots showing the conformational states of PARP$$ when interacting with different compounds. (**a**) Interaction with DK1. (**b**) Interaction with EK2.

To determine whether the interaction of naturally occurring and synthetic secondary metabolites affects the structure of the PARP-1 protein, we performed a Ramachandran diagram analysis; here, we discuss the best synthetic and naturally occurring metabolites. Thus, in the case of the interaction with DK1 (see Figure 11a), we find that seven hundred fifty-three residues were found in favored regions; in the additional allowed regions, we found a total of one hundred and thirty-three residues, and in the generously allowed regions, we found six amino acid residues. Only five amino acids were found in the not-permitted regions, making for a total of eight hundred and ninety-seven amino acids. For this, several glycines and prolines have been excluded; seventy-one in the case of glycines and forty-four in the case of prolines. It should be noted that, among the favored, allowed, and generously allowed regions, the residues present lead to total of 99.4, which tells us that this protein exists in nature in its complex form if feasible.

In the case of PARP-1 interacting with EK2, the Ramachandran analysis of this is presented in Figure 11b, from which we can see that seven hundred and forty-five amino acids are found in favored regions and one hundred and forty-four are found in favored areas. There are a further eight in additionally allowed and generously allowed regions, making for a total of eight hundred and ninety-seven amino acids; as in all cases of the Ramachandran analysis, the final residues were excluded, as were all glycines and prolines—seventy-one in the case of glycines and forty-four in the case of prolines. Considering the favored, additionally allowed, and generously allowed regions, we find that these residues make up a total of 100%, from which we can conclude that this type of interaction favors the formation of secondary structures, indicating that this complex system very probably exists in nature.

## 4. Conclusions

The present study shows that synthetic compounds have a better affinity with PARP-1 than those of natural origin investigated in this article. Still, the energetic difference between both groups is approximately 11.00 kcal/mol. We believe that these results lead us to assume that, in the absence of synthetic compounds, PARP-1 can and does react with compounds of natural origin; this gives us an indication that, in the presence of compounds of natural origin, the PARP-1 protein, when inhibited, stops fulfilling its replication function, leading the cell to a condition of apoptosis. The compounds of natural origin investigated in the present study come from the following plants used for medicinal purposes: Astilbe chinensis (EK1), Scutellaria barbata D. Don (EK2), Uncaria rhynchophylla (EK3), Fallugia paradoxa (EK4), and Curcuma zedoaria (Christm.) Rosc. (EK5).

## Figures and Tables

**Table 1:** The binding energies for each of the interacting systems ^1^.

Protein	Inhibitor	Energy
PARP-1	DK1	−9.999
	DK2	−9.573
	DK3	−9.294
	DK4	−7.916
	DK5	−10.260
PARP-1	EK1	−9.997
	EK2	−9.924
	EK3	−9.182
	EK5	−9.520
	EK5	−8.209

^1^ For synthetic and natural origin systems that interacted with PARP-1, in kcal/mol.

**Table 2:** Binding energies from the MMPBSA analysis.

Molecule	Binding Energy ^1^
DK1	−99.927 ± 6.615
DK2	−65.858 ± 9.097
DK3	−63.680 ± 20.180
DK4	−82.934 ± 6.662
DK5	−67.150 ± 6.825
EK1	−65.414 ± 8.264
EK2	−88.301 ± 6.897
EK3	−77.123 ± 6.562
EK4	−40.123 ± 8.044
EK5	−55.813 ± 5.506

^1^ In kcal/mol.

## Data Availability

Data is contained within the article.
